# The Activity of 1,8-Dihydroanthraquinone Derivatives in Nervous System Cancers

**DOI:** 10.3390/molecules29245989

**Published:** 2024-12-19

**Authors:** Estera Okoń, Wirginia Kukula-Koch, Agata Jarząb, Katarzyna Gaweł-Bęben, Ewelina Bator, Magdalena Michalak-Tomczyk, Jacek Jachuła, Beata Antosiewicz-Klimczak, Adrian Odrzywolski, Wojciech Koch, Anna Wawruszak

**Affiliations:** 1 Department of Biochemistry and Molecular Biology, Medical University of Lublin, 1 Chodzki Str., 20-093 Lublin, Poland; estera.okon@umlub.pl (E.O.); agata.jarzab@umlub.pl (A.J.); adrian.odrzywolski@umlub.pl (A.O.); anna.wawruszak@umlub.pl (A.W.); 2 Department of Pharmacognosy with Medical Plants Garden, Medical University of Lublin, 1 Chodzki Str., 20-093 Lublin, Poland; virginia.kukula@gmail.com; 3 Department of Cosmetology, University of Information Technology and Management in Rzeszów, 2 Sucharskiego, 35-225 Rzeszów, Poland; kagawel@wsiz.edu.pl (K.G.-B.); bantosiewicz@wsiz.edu.pl (B.A.-K.); 4 Interdisciplinary Center for Preclinical and Clinical Research, Rzeszow University, 2a Werynia, 36-100 Kolbuszowa, Poland; ebator@ur.edu.pl; 5 Department of Physiology and Toxicology, The John Paul II Catholic University of Lublin, 1I Konstantynów Str., 20-708 Lublin, Poland; magdalena.michalak@kul.pl; 6 Department of Botany, Mycology and Ecology, Institute of Biological Sciences, Maria Curie-Skłodowska University, 19 Akademicka Str., 20-033 Lublin, Poland; jacek.jachula@mail.umcs.pl; 7 Department of Food and Nutrition, Medical University of Lublin, 4a Chodzki Str., 20-093 Lublin, Poland

**Keywords:** nervous system cancers, natural products, biological activity, 1,8-dihydroanthraquinone derivatives, emodin, aloe-emodin, hypericin, chrysophanol, rhein, physcion

## Abstract

Primary and metastatic tumors of the nervous system represent a diverse group of neoplasms, each characterized by distinct biological features, prognostic outcomes, and therapeutic approaches. Due to their molecular complexity and heterogeneity, nervous system cancers (NSCs) pose significant clinical challenges. For decades, plants and their natural products with established anticancer properties have played a pivotal role in the treatment of various medical conditions, including cancers. Anthraquinone derivatives, a class of tricyclic secondary metabolites, are found in several botanical families, such as *Fabaceae*, *Polygonaceae*, *Rhamnaceae*, and *Rubiaceae*. In a comprehensive review, recent advancements in the anticancer properties of 1,8-dihydroanthraquinone derivatives—such as emodin, aloe-emodin, hypericin, chrysophanol, rhein, and physcion—were analyzed. These compounds have been studied extensively, both used individually and in combination with other chemotherapeutic agents, using in vitro and in vivo models of nervous system tumors. It was demonstrated that 1,8-dihydroanthraquinone derivatives induce apoptosis and necrosis in cancerous cells, intercalate into DNA, disrupting transcription and replication in rapidly dividing cells, and alter ROS levels, leading to oxidative stress that damages tumor cells. Additionally, they can influence signaling pathways involved in oncogenesis, such as MAPK, PI3K/Akt, or others crucial for the survival and the proliferation of NSC cells. The exploration of 1,8-dihydroanthraquinone derivatives aims to develop novel therapies that could overcome resistance and improve cancer patients’ outcomes.

## 1. Introduction

Nervous system cancers (NSCs), which include primary tumors of the brain and spinal cord as well as metastatic tumors, present complex challenges in both diagnosis and treatment. These cancers are characterized by their unique biology as well as their diverse clinical manifestations. The development of NSCs is driven by a combination of genetic mutations, epigenetic alterations, and aberrant signaling pathways [1]. Understanding the biological landscape of NSC and its phenotypic heterogeneity is crucial for developing novel anticancer therapies. Despite extensive research, the precise pathogenesis of NSC remains unclear, and the effective treatment of this disease, due to the complexity of tumor biology, the protective mechanisms of the brain (e.g., blood–brain barrier), strong diverse effects, and the inherent resistance to conventional therapies, is a significant challenge in modern medicine [2]. There is ongoing research aimed at identifying new naturally derived compounds with lower toxicity profiles. The search for these compounds is driven by the need to discover therapeutic agents that retain efficacy while minimizing harmful side effects commonly associated with synthetic drugs. Ultimately, the development of naturally derived compounds with reduced toxicity has the potential to revolutionize treatment paradigms, offering safer and more effective alternatives to traditional therapies, particularly in the treatment of complex diseases such as cancer [3].

Bioactive substances of natural origin can influence the process of carcinogenesis by modulating the behavior of neoplastic cells and targeting signaling pathways that are aberrantly activated or suppressed [4]. Anthracene and its derivatives represent a significant class of compounds that have been extensively studied in recent years. Anthraquinones, a diverse class of secondary metabolites in plants, are known for their significant structural diversity, pronounced biological activity, and low toxicity. These compounds are predominantly found in plant families such as *Fabaceae*, *Liliaceae*, *Polygonaceae*, *Rubiaceae*, *Rhamnaceae*, and *Scrophulariaceae* [4]. The compounds exhibit a wide range of biological activities, including anti-inflammatory [5], immunomodulatory [6], wound-healing [7], and antineoplastic properties [8].

Therefore, this manuscript provides a comprehensive review of the latest advancements in the anticancer activities of 1,8-dihydroanthraquinones, including emodin, aloe-emodin, hypericin, chrysophanol, rhein, and physcion. These compounds have been evaluated both individually and in combination with other chemotherapeutic agents in in vitro and in vivo models ofNSCs.

## 2. The Molecular and Genetic Basis of Neurological Tumors

Neurological tumors encompass cancers that originate in the central or peripheral nervous system. Their classification is primarily based on the main cell types from which they arise, typically determined through morphological characteristics and immunohistochemical analysis [9].

**Figure 1:**
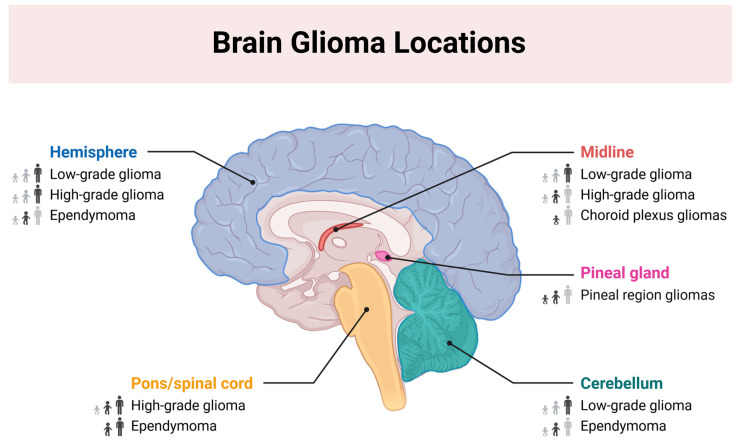
Brain glioma locations. This figure was created with BioRender.com (Toronto, ON, Canada) (accessed on 26 November 2024 to A.W.).

Most neurological tumors originate from glial cells. These tumors, known as gliomas, include tumors primarily composed of astrocytes (astrocytoma), oligodendrocytes (oligodendroglioma), and ependymal cells (ependymoma) (Figure 1) [9].

**Figure 2:**
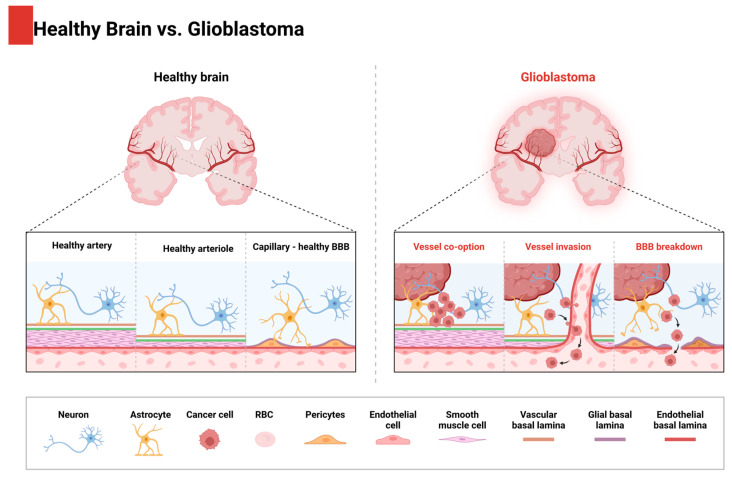
The morphological differences between a healthy brain and glioblastoma. This figure was created with BioRender.com (Toronto, ON, Canada) (accessed on 26 November 2024 to A.W.).

Astrocytoma is the most common type of central nervous system tumor, accounting for over 60% of all primary brain tumors. The most malignant form of infiltrating astrocytic tumors is glioblastoma multiforme (GBM), which is one of the most aggressive cancers, characterized by a very short survival time, with an average of less than one year (Figure 2) [9].

It is important to differentiate between various types of gliomas based on mutations in isocitrate dehydrogenase (IDH) 1 or 2. The most common mutation in these genes is a single amino acid change, where histidine replaces arginine. This alteration leads to the creation of a new enzyme function, which converts alpha-ketoglutarate into D-2-hydroxyglutarate. The result of this process is abnormal deoxyribonucleic acid (DNA) and histone methylation, leading to the widespread hypermethylation of CpG islands [10]. *IDH* mutations often occur in conjunction with mutations in p53, one of the most important tumor suppressor proteins, which is associated with nearly all types of cancer, including gliomas. Alterations in the p53 pathway, particularly in low-grade gliomas, are thought to promote progression to more aggressive forms. In primary glioblastomas, there is often loss of the *INK4A/ARF* (cyclin-dependent kinase inhibitor 2A/B—*CDKN2A*) gene locus, mutations in the phosphatase and tensin homolog deleted on the chromosome 10 (*PTEN*) gene, and amplification or loss of the epidermal growth factor receptor (*EGFR*) gene (Table 1) [11].

Secondary glioblastomas more frequently exhibit direct mutations in the p53 gene. Another key element in gliomas is the amplification of the platelet-derived growth factor receptor alpha (PDGFRα), which primarily activates the Ras/Raf/MAPK pathway. This pathway regulates the activity of transcription factors that influence processes such as cell proliferation, survival, differentiation, and apoptosis [11].

Oligodendrogliomas are diffuse gliomas characterized by IDH mutations and the loss of chromosomes 1p and 19q (1p/19q codeletion) [12], which allows them to be classified as IDH-mutant and 1p/19q-codeleted oligodendrogliomas. For this reason, all diffuse gliomas with IDH mutations should be tested for 1p/19q codeletion. However, an IDH-mutant oligodendroglioma can also be diagnosed without testing for 1p/19q if immunohistochemical analysis reveals the clear loss of ATRX expression and/or the diffuse expression of TP53. Typical mutations in oligodendrogliomas also include those in the *TERT* promoter region (Table 1) [12].

Pilocytic astrocytomas (PAs) were recognized as a distinct clinical entity several decades ago. These are relatively benign tumors, characterized by an approximately 10-year survival period. For a long time, little was known about the molecular mechanisms underlying their development. It was only with the use of modern high-throughput sequencing techniques that examine the entire genome that it was revealed that nearly all cases involve single abnormalities in the mitogen-activated protein kinase (MAPK) signaling pathway [13].

Pleomorphic xanthoastrocytoma (PXA) is a rare tumor, accounting for less than 1% of all astrocytomas. It primarily occurs in younger patients and generally has a favorable prognosis. However, it exhibits variable morphology, which is associated with the potential for recurrence and spread within the central nervous system. Recent advances in molecular research have revealed that the V600E mutation in the *BRAF* gene in combination with *CDKN2A/B* deletions and telomerase reverse transcriptase (*TERT*) promoter mutations are the most common genetic alterations in PXA. These tumors can present diagnostic challenges because their histopathological and genetic profiles overlap with other tumor types, particularly epithelial gliomas [14].

Subependymal giant cell astrocytoma (SEGA) most commonly occurs in patients with tuberous sclerosis complex (TSC). Although it is classified as a benign tumor, it can pose a serious health risk, leading to severe complications, including death in young patients. Surgery and chemotherapy are standard treatment methods. The preoperative use of mTOR inhibitors for SEGA tumors located in deep, hard-to-reach areas of the brain helps reduce their size, facilitating complete resection [15].

Ependymomas are rare neuroectodermal tumors that arise from ependymal cells. Data regarding their cytogenetic features are limited. The most common abnormalities include an incorrect number of copies of chromosome 22 and various translocations of chromosome 22, which occur in about 30% of cases. Ependymomas differ genetically from astrocytic and oligodendroglial tumors. No mutations or deletions of tumor suppressor genes, such as cyclin-dependent kinase 2 inhibitor B (*CDKN2B*) and cyclin-dependent kinase 2 inhibitor A (*CDKN2A*), or amplifications of cyclin-dependent kinase 4 (*CDK4*), *CCND1*, or *EGFR* have been observed. The role of the *p53* gene is marginal and not fully understood. In contrast, amplification of the mouse double minute 2 homolog (*MDM2*) gene is present in 35% of ependymoma cases. Cytogenetic aberrations vary depending on the patient’s age and tumor location. Intraventricular ependymomas often present with headaches, nausea, vomiting, and dizziness, which result from increased intracranial pressure and hydrocephalus [16].

Tumors of the peripheral nervous system form a diverse group with a wide range of morphological characteristics. They include both benign and treatable tumors, such as schwannomas, as well as those that are benign but may exhibit local aggressiveness, such as plexiform neurofibromas. This group also includes highly malignant tumors, such as malignant peripheral nerve sheath tumors (MPNSTs) [17]. The most common types include schwannomas and neurofibromas. Schwannomas are benign tumors that originate from Schwann cells of the peripheral nerve sheath, often involving the vestibular nerve. Malignant transformation is rare. In addition to inactivation of the neurofibromatosis type II (*NF2*) gene in schwannomas, other genes are involved in their pathogenesis, such as leucine zipper-like post-translational regulator 1 (*LZTR1*), the SWI/SNF-related matrix-associated actin-dependent regulator of chromatin subfamily B member 1 *(SMARCB1*), and coenzyme Q6 monooxygenase (*COQ6*). Understanding the molecular mechanisms allows for targeted genetic therapy, especially in the case of vestibular schwannomas, using mTOR inhibitors (rapamycin), EGFR inhibitors (lapatinib), or VEGF inhibitors (bevacizumab) [18].

Meningiomas, or tumors of the meninges, develop from the arachnoid layer of the meninges and are histologically classified into three grades: benign, atypical, and malignant. Meningiomas exhibit epigenetic changes, such as the hypermethylation of tumor suppressor genes like p73 in grade I tumors and genes like *TIMP3*, maternally expressed 3 *(MEG3*), the glutathione S-transferase pi gene *(GSTP10*, homeobox A6 *(HOXA6*), homeobox A9 *(HOXA9*), WNK lysine deficient protein kinase 2 *(WNK2*), and *UPK3A*, which occur with increasing frequency depending on the grade of malignancy. The aberrant expression of genes associated with the IGF and Wnt signaling pathways is linked to disease progression. In summary, meningiomas show extensive genetic changes that may aid in prognostic assessment [19].

Interactions between the nervous system and cancer significantly influence processes such as oncogenesis, tumor growth, invasion, metastasis, treatment resistance, and immune suppression against tumors. Advances in the neurobiology of cancer hold the potential to become a crucial component of modern therapeutic strategies in oncology [20]. Advanced research in the field of NSCs is essential to develop more effective and safer therapeutic approaches, aiming to improve outcomes for individuals diagnosed with these aggressive neoplasms. Numerous ongoing studies are focused on exploring alternative treatment options, including natural products. Among the promising candidates with demonstrated anticancer properties are 1,8-dihydroanthraquinone derivatives.

## 3. Characteristics of Anthracene Derivatives

**Figure 3:**
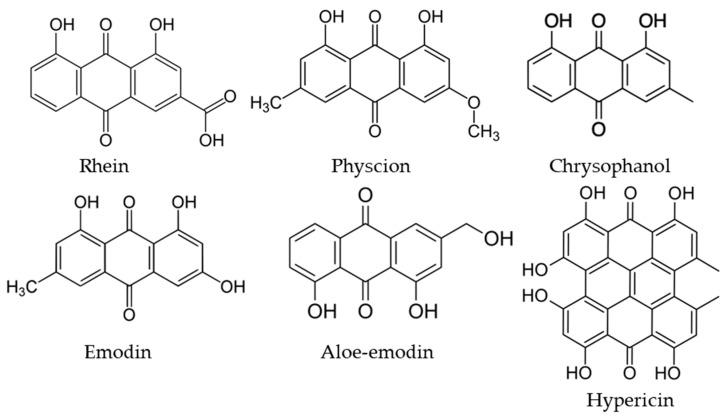
Chemical structures of anthracene derivatives (aloe-emodin, emodin, chrysophanol, hypericin, physcion, rhein) are described in this manuscript.

Anthracene derivatives, particularly anthraquinones (Figure 3), are among the most extensively studied natural products due to their broad spectrum of biological activities, including antifungal, antibacterial, antioxidant, anticancer, anti-inflammatory, and laxative properties. Over 81% of plants containing anthraquinones belong to three botanical families: *Polygonaceae*, *Rubiaceae*, and *Fabaceae*. However, they are also found in families such as *Liliaceae*, *Rhamnaceae*, and *Scrophulariaceae* [21]. These natural compounds have been known and widely utilized for centuries, particularly in ancient Persia, Egypt, and India, where they were used as medicinal agents, natural dyes (e.g., alizarin), and even as food additives. In recent years, their anticancer properties have attracted significant attention due to their high efficacy in treating cancers such as breast, lung, prostate, and intestinal cancers, along with their relatively low toxicity [8].

Anthracene derivatives are plant secondary metabolites containing aromatic hydrocarbon consisting of three linearly fused benzene rings [22]. Anthrons and anthranols are the most common forms of reduced anthracene derivatives, which are mainly present in fresh plant material, whereas anthraquinones are the most common oxidized form, which is mainly synthetized during long storage or drying. Most of these compounds are derivatives of 9,10-anthracenedione, formed by a basic anthracene moiety, containing two ketone groups at the positions 9 and 10. Among them, anthraquinones are the most important one, characterized by a large structural diversity and wide spectrum of applications [23]. So far, over 700 compounds from this group have been identified, and the plants richest in these compounds are various species of the genera *Rheum*, *Rumex*, *Polygonum*, *Aloe* and *Cassia* [8]. Around 200 of these natural substances have been identified in flowering plants, while the rest were issued from lichens and fungi. They can be found in all parts of the plants, like roots, rhizomes, leaves, fruits, and flowers [23]. Emodin, rhein, physcion, catenarin, chrysophanol, and aloe-emodin are the most frequently reported aglycone anthraquinones. Glycosylated forms can also be found, e.g., in rhizome to favor their accumulation and storage in plants, but they are decomposed by β-glucosidases or oxidative processes to aglycone forms; therefore, some studies have indicated the presence of only non-glycosylated forms in varied seeds [24,25]. Chemical structures of the main anthracene derivatives frequently observed in natural products are shown in Figure 3.

Due to their wide spectrum of biological activities, anthraquinones are extensively studied for their physiological properties, chemical synthesis, extraction techniques from natural sources, and their associated risks and health benefits to humans [23]. Many natural anthraquinones have been chemically modified, and the resulting semi-synthetic products are now widely used as approved anticancer drugs. Anthracycline antibiotics, initially isolated from *Streptomyces peucetius* var. *caesius*, such as doxorubicin and daunorubicin, along with chemically modified derivatives like valrubicin, mitoxantrone, and pixantrone, are widely employed as anticancer agents. These compounds are used in the treatment of both hematological and solid malignancies, including acute lymphocytic leukemia, Hodgkin’s lymphoma, bladder cancer, breast cancer, and various other metastatic cancers [26].

## 4. Activity of 1,8-Dihydroanthraquinone Derivatives in In Vitro Models of Nervous System Cancers

### 4.1. Emodin and E8OG

Emodin is known to target the aryl hydrocarbon receptor (AhR) [27], a ligand-activated transcription factor, expressed widely in various species and tissues. The AhR is also activated by other natural compounds, including resveratrol, curcumin, or carotene [28,29,30]. Upon activation, it translocates to the nucleus, forms a heterodimer with the aromatic hydrocarbon receptor nuclear transporter (ARNT), and binds to the corresponding dioxin response element (DRE), followed by the activation of several downstream genes regulating toxicological and biological responses [31]. The expression of the AhR increases in invasive tumors and has higher nuclear localization than in noncancerous tissue, which indicates that the AhR signaling pathway is active and related to the malignant phenotype of tumor cells. The activation of AhR by different ligands leads to different biological responses, resulting in either promotion or suppression of the progression of cancer [32]. In glioblastoma cells, activation of the AhR by emodin in low concentrations (10^−6^–10^−7^ M) inhibits cell migration in a time-dependent manner. At higher concentration (10^−5^–10^−6^ M), emodin significantly decreased the viability of glioblastoma cells by >70% [33]. Necroptosis is one of the most important mechanisms of programmed cell death, and emodin could induce this process in glioma, possibly through the activation of the TNF-α/RIP1/RIP3 axis (Table 2) [34].

Emodin was found to be a potent inhibitor of phosphoglycerate mutase 1 (PGAM1) and inhibited the enzyme activity by 50% (IC_50_) at 19.82 μM in U-87 malignant glioma cells or 17.08 μM in patient-derived X01 cells. PGAM1 catalyzes the conversion of 3-phosphoglycerate (3-PG) to 2-PG in glycolysis, and it was shown using 3D cultures and wound-healing assays that the glycolysis inhibition by emodin effectively blocked the radiation-induced aggressiveness of U87 glioblastoma cells [35]. In glioblastoma cells, emodin also acts as a selective inhibitor of protein kinase CKII (casein kinase II), a ubiquitous and highly conserved Ser/Thr kinase that plays a critical role in cell proliferation and oncogenesis [36]. Emodin also suppressed the PMA-induced and basal activity of phospholipase D (PLD), catalyzing the hydrolysis of phosphatidylcholine (PC), the major membrane phospholipids, to form phosphatidic acid (PA) and choline. PLD was recently shown to be activated (at least in part) by CKII [37].

Emodin can also effectively inhibit HA-induced matrix metalloproteinase (MMP) secretion and the invasion of glioma through inhibition of the focal adhesion kinase (FAK), the extracellular signal-regulated kinases 1/2 (ERK1/2), protein kinase B (Akt/PKB) activation and partial inhibition of the activator protein-1 (AP-1), and the nuclear factor kappa-light-chain-enhancer of activated B cell (NF-kappaB) transcriptional activities. The treatment of emodin suppressed not only the HA-induced MMP-9 secretion, but also the constitutive MMP-2 secretion in a concentration-dependent manner. Emodin suppressed the expression of the extracellular matrix (ECM) degrading proteases, MMP-2, and MMP-9 at the transcriptional level [38].

In the C6 mouse glioma model, emodin was shown to have biphasic activity, with the initial pro-apoptotic effects and later development of drug resistance [39]. Emodin treatment increased the expression and activation of pro-apoptotic p53, Bax, B-cell lymphoma-2 (Bcl-2), Fas, and caspase-3. Following 24 h treatment, emodin decreased NF-kappaB expression, but following 48 h of exposure, emodin increased NF-kappaB activity and induced NF-kappaB translocation from cytoplasm to nuclei. Emodin was also shown to initially reduce p-JNK and p-p38 levels (following 12 to 24 h of treatment), but after longer exposure times (48 and 72 h of incubation), the levels of p-JNK and p-p38 were increased.

By inhibiting the activation of nuclear factor-kappa B (NF-κB), emodin is also involved in the suppression of malignant phenotypes of glioma cells. The treatment of U251 glioma cells with emodin significantly decreased mRNA and protein expression levels of NF-KB-regulated expressions of syndecan-1, a surface heparan sulfate that proteoglycan presents in malignant glioma cells but not in normal brain specimens [40].

The interactions between syndecans and thrombospondin-1 secreted by malignant glioma cells is a mechanism that increases the motility of glioma cells [40]. By means of fluorescence microscopy, the intracellular distribution of emodin was studied. The selective photoactivation of emodin in a precisely defined position (nuclear envelope) allowed moderately hydrophobic emodin to enter the nucleus. The redistribution of quinizarin, emodin, and hypericin (Hyp) between lipids, proteins, and DNA were studied in solutions and cells. Based on these results, a theoretical model of hydrophobic drugs’ nuclear internalization after photo-activation was proposed. The presence of 10 μM quinizarin, emodin, or Hyp increased P-glycoprotein function in U87 MG cells. Moreover, emodin pretreatment allowed quinizarin nuclear internalization without photo-activation, which was not the case for Hyp [41].

Interesting results were also obtained from in vitro experiments involving glioma stem cells (GSCs). Emodin strongly inhibited the self-renewal capacity of GSCs, inhibited the stemness of GSCs by blocking the interaction of EGFR/EGFRvIII with Hsp90, which led to the degradation of EGFR/EGFRvIII followed by the suppression of stemness signaling pathways involving Notch, β-catenin, and STAT3. In addition, emodin partially induced apoptosis, inhibited invasion, and sensitized GSCs to IR [42].

The long-time use of emodin may cause damage to the liver, kidneys, and the reproductive system. One way to reduce the toxicity of emodin and improve its chemical properties and biological activity is the synthesis of glycosylated derivatives, such as emodin-8-O-glucoside (E8OG) [43]. E8OG is also known for its ability to cross the blood–brain barrier (BBB) [44], and, therefore, it is a promising therapeutic agent for various types of brain cancers. E8OG, isolated from Reynoutria japonica Houtt, was shown as an antiproliferative and cytotoxic agent towards SK-N-AS neuroblastoma, T98G human glioblastoma, and C6 mouse glioblastoma cancer cells, with IC_50_ values of 108.7 µM, 61.24 µM, and 52.67 µM, respectively [4].

The SH-SY5Y human neuroblastoma cell line was also used in several studies investigating neuroprotective effects of emodin towards various chemical factors. One of such studies showed that emodin alleviates H_2_O_2_-induced apoptosis and neuroinflammation potentially by influencing the PI3K/mTOR/GSK3β signaling pathway. In this study, emodin in a concentration range from 10 to 100 µM increased the viability of the H_2_O_2_-treated cells following 24 h treatment, decreased apoptosis and LDH release. In H_2_O_2_-treated SH-SY5Y cells, emodin downregulated the release of inflammatory factors IL-6, NO, and TNF-α, decreased the protein levels of H_2_O_2_-induced oxidative stress markers (COX-2 and iNOS), and reversed mitochondrial dysfunction, measured by ATP, NAD+ and cytochrome C levels [45]. In another study, pre-treatment with emodin suppressed the effects of H_2_O_2_ on the activity of the mitochondrial complexes I and V, as well as on the production of adenosine triphosphate (ATP) and on the mitochondrial membrane potential (MMP). Emodin also prevented the H_2_O_2_-induced disruption of the tricarboxylic acid cycle (TCA). Emodin treatment was also able to decrease the secretion of proinflammatory interleukin-1β (IL-1β) and tumor necrosis factor-α (TNF-α) by the H_2_O_2_-treated cells. Inhibition of the adenosine monophosphate-activated protein kinase (AMPK) or silencing of the transcription factor nuclear factor erythroid 2-related factor 2 (Nrf2) abolished the protection induced by emodin in the H_2_O_2_-challanged SH-SY5Y cells [46].

Emodin also showed a neuroprotective effect on SH-SY5Y cells treated with high concentrations of ZnSO_4_ (300 µM). Zinc is an essential trace element important for the physiological function of the central nervous system, but its accumulation inside neurons may induce oxidative stress and mitochondrial dysfunction. These changes contribute to many brain diseases [47]. Emodin ameliorated the zinc-induced altered expression of levels of phosphorylated ERK1/2 and synaptic proteins (presynaptic SNAP 25, synaptophysin, and postsynaptic PSD95). Moreover, emodin inhibited the generation of reactive oxygen species and oxidative stress and facilitated the collapse of mitochondrial membrane potential (ΔΨm) in SH-SY5Y cells [48]. Pre-treatment with emodin also decreased the intracellular Zn+ levels in SH-SY5Y cells, enhanced cell viability, and reduced cell apoptosis and lactate dehydrogenase release. Further in-depth investigations showed that emodin decreased the expression levels of zinc transporter-1, metallothionein-1, and metallothionein-2 as well as prevented the depletion of NAD+ and ATP induced by zinc. Emodin also reduced intracellular reactive oxygen species and endoplasmic reticulum stress levels. Thus, emodin could protect against neurotoxicity induced by Zn^2+^ in neuroblastoma SH-SY5Y cells [49].

Another study using the SH-SY5Y cell line investigated neuroprotective effects of emodin towards synaptic proteins and oxidative stress damage caused by high doses of NaF. Emodin pre-treatment significantly recovered several alternations induced by NaF, including increased levels of p-ERK1/2, decreased expressions of Nrf2 and HO-1, and elevated the production of ROS, 4-hydroxynonenal (4-HNE), and 8-Hydroxy-2′-deoxyguanosine (8-OHdG) [50].

Anthraquinone derivatives, including emodin and chrysophanol, were also evaluated for their anticancer potential using molecular docking. Binding interactions and energy profiles for emodin and chrysophanol with four major cancer-related protein targets were analyzed. Docking studies indicated that chrysophanol had higher binding free energy compared to emodin [51].

**Table 2:** Anticancer activity of emodin and its derivatives in in vitro models of nervous system cancers. (AhR—aryl hydrocarbon receptor; EGFR/EGFRvIII—epidermal growth factor receptor/EGFR variant III; E8OG—emodin-8-O-glucoside; CKII—casein kinase II; PLD—phospholipase D; α-KGDH—α-ketoglutarate dehydrogenase; SDH—succinate dehydrogenase; 4-HNE—4-hydroxynonenal; 8-OHdG—8-Hydroxy-20-deoxyguanosine; SNAP25—synaptosome associated protein 25; PSD95—postsynaptic density 95; ZnT-1—zinc transporter-1; MT1—metallothionein-1; MT2—metallothionein-2; and AMPK—AMP-activated protein kinase).

Compound	Model	Cell Line	Mechanism of Action	Dose	References
Emodin	In vitro monotherapy	U87MG astroglioma	↓ proliferation ↓ activation of CKII and PLD	50 µM (24 h)	[37]
Emodin	In vitro monotherapy	U87MG, U373MG, U251MG, glioblastoma and HS683 glioma cell lines	↓ apoptosis ↓ migration and invasion	20 µM	[38]
Emodin	In vitro monotherapy	C6 mouse glioblastoma	initial apoptosis-induced cell death, but later effects resulting in drug resistance	30 µM	[39]
Emodin	In vitro monotherapy	U251 human malignant glioblastoma	↓ cell proliferation and viability ↑ apoptosis and necroptosis ↑ number of cells G0/G1 ↑ the ratios of cells in the S and G2/M phases	10 μM (12 h) IC_50_ = 22.44 μM	[34]
Emodin	In vitro monotherapy	Glioma stem cells (GSCs)	↑ apoptosis ↓ cell invasiveness ↑ sensitivity to ionizing radiation ↓ expression of Notch intracellular domain, nonphosphorylated β-catenin, and phosphorylated STAT3 protein ↑ proteosomal degradation of EGFR/EGFRvIII	5 μM (24 h or 48 h)	[42]
Emodin	In vitro monotherapy	LN18 and LN428 glioblastoma cells	↑ radiosensitivity to γ-radiation ↑ autophagy and apoptosis in response to neutron radiation ↓ cell migration and invasiveness in response to neutron radiation		[52]
Emodin	In vitro monotherapy	U87 glioblastoma	↓ viability ↓ cell migration ↑ AhR signaling pathway (↑ CYP1B1 and IL-24 expression)	10^−5^ M–10^−6^ M (24 h, 48 h) 10^−6^ M–10^−7^ M (12 h, 24 h, 36 h)	[33]
Emodin	In vitro monotherapy	U251 malignant glioma	↓ NF-κB activation ↓ syndecan-1 mRNA and protein levels	25 or 50 µg/mL (16 h)	[40]
Emodin	In vitro monotherapy	U87 human glioma	↓ E-cadherin, fibronectin, and vimentin expression ↓ cell invasiveness	20 µM	[35]
Emodin	In vitro monotherapy	SH-SY5Y human neuroblastoma	↓ proliferation ↓ invasion and migration ↓ expression of GRB2, RhoA, HIF-1a, VEGF, FAK, iNOS, COX2, p-p38, p-c-jun, MMP2, MMP9, and MMP7 ↑ expression of PKC, PI3K, MEKK3, and NF-jB p65	25 µM (IC_50_ for 24 h treatment)	[53]
Emodin	In vitro monotherapy	IMR-32 human neuroblastoma	↑ apoptosis ↑ activation of caspase-2 and 9 ↑ p53 and p21 expression ↑ intracellular ROS, Ca and NO levels ↓ mitochondrial membrane potential	10 and 20 µM (24 h)	[54]
E8OG	In vitro monotherapy	SK-N-AS neuroblastoma	↑ Cytotoxicity ↓cell division	IC_50_ = 108.7 µM (96 h)	[4]
		T98G human glioblastoma		IC_50_ = 61.24 µM (96 h)	
		C6 mouse glioblastoma		IC_50_ = 52.67 µM (96 h)	
Emodin	In vitro monotherapy	SH-SY5Y human neuroblastoma treated with H_2_O_2_	↑ cell viability ↓ apoptosis and LDH release ↓ release of IL-6, TNFα and NO ↓ COX-2 and iNOS protein expression ↓ inracellular ROS ↑ ATP and NAD+ levels ↓ cytochrome C expression ↓ activation of PI3K/mTOR/GSK3*β* signaling pathway	10–100 µM	[45]
Emodin	In vitro monotherapy	SH-SY5Y human neuroblastoma treated with H_2_O_2_ (300 μM)	↑ cell viability ↓ cytotoxicity ↓ ROS production ↓ mitochondrial lipid reoxidation, protein carbonylation, and protein nitration, ↑ activity of aconitase, α-KGDH, and SDH, ↑ activity of mitochondrial complexes I and V ↓ IL-1β and TNFα release ↓ NF-κB activation	40 µM (4 h pre-treatment)	[46]
Emodin	In vitro monotherapy	SH-SY5Y 26 human neuroblastoma treated with ZnSO_4_ (300 µM)	↓ cell death ↓ p-ERK1/2 ↑ loss of mitochondrial potential ↓ intracellular ROS	10, 25 and 40 μM	[48]
Emodin	In vitro monotherapy	SH-SY5Y 26 human neuroblastoma treated with ZnSO_4_ (200 µM)	↑ cell viability ↓ cell apoptosis and LDH release ↓ intracellular Zn^+^ levels ↓ expression of ZnT-1, MT1 and MT2 ↑ NAD+ and ATP ↓ inracellular ROS ↓ activation of AMPK	10–50 µM (1 h pre-treatment)	[49]
Emodin	In vitro monotherapy	SH-SY5Y human neuroblastoma treated with NaF (24 h, 50 ppm)	↑ protein expression of SNAP25, synaptophysin, and PSD95 ↓ p-ERK1/2 ↑ Nrf2 and HO-1 ↓ ROS, 4-HNE, and 8-OHdG	10, 25, and 40 μM (2 h pre-treatment)	[50]

### 4.2. Aloe-Emodin

The results of recent studies indicate that aloe-emodin (AE) (Table 3) may have a protective effect against the reperfusion of bilateral carotid artery (MO/RCA) damage, likely due to its antioxidant properties. The inhibitory effect of AE on cerebrotoxic proteins involved in apoptosis and neuroinflammation was confirmed through in silico docking analysis. Previous research included the molecular docking of AE with proteins involved in apoptosis (Bax and CASP3) and inflammation (TNF-α, IL-6, ASIC, and GR) to evaluate binding affinity based on binding energy and interactions. These neurotoxic proteins are upregulated during neuroinflammation, and ASIC contributes to neuronal damage through acidosis. Docking results indicated that AE interacted with the active sites of TNF-α (VAL A:17, 150, ALA A:17, 18, 33, PRO A:20, ARG A:32), CASP3 (HIS A:121, ARG B:207, TRP A:414, CYC A:420), NOS (PHE A:589), and GR (chain A: THR501, TYR753, GLU726, TYR471). It is suggested that AE may interfere with TNF-α, CASP3, NOS, and GR function through hydrogen bonding and hydrophobic interactions, potentially inhibiting disease progression [55].

Bax plays a key role in managing the proliferation of cancer cells by initiating apoptosis. Designing new inhibitors with higher specificity and efficacy for Bax remains a major challenge in cancer therapy. AE has been shown to inhibit tumor growth by triggering apoptosis. The molecular docking of 20 AE-based derivatives revealed variations in their binding mechanisms, linked to differences in chemical structures. Key amino acid residues such as Lys58, Cys62, and Leu162 were identified as critical binding sites. Among the tested compounds, AE-15 exhibited the strongest binding affinity to Bax, although further research is required to validate its therapeutic potential [51].

Common commercial GBM cell lines like U87MG, U251, T98G, and A172 are genetically distant from primary tumors due to frequent passages in culture, resulting in a loss of the typical heterogeneity of GBM cells. Additionally, the methylated MGMT gene promoter in U87MG cells makes it unsuitable for studying drug resistance in vitro. In contrast, in recent studies, the primary GBM cell lines NULU and ZAR were used, which have a non-methylated MGMT promoter and display a drug-resistant phenotype, closely resembling patient tumors. This model allowed the investigation of resistance mechanisms to TMZ (temozolomide) treatment. It assessed the effects of AE (20 µM), both alone and combined with TMZ (10 µM), on cell growth and viability at 24, 48, and 72 h post-exposure in the NULU and ZAR lines. Results showed that AE reduced cell growth and viability as early as 24 h, with a stronger effect when combined with TMZ, suggesting that AE enhances TMZ’s efficacy. Moreover, the molecular characterization of NULU and ZAR cells confirmed the non-methylated MGMT gene promoter, a key indicator of TMZ resistance, since cells with high MGMT activity can mitigate the cytotoxic effects of alkylating agents like TMZ. Additionally, the study explored the role of the NF-κB signaling pathway, which is implicated in GBM treatment resistance. Western blot analyses of MGMT and NF-κB protein levels after AE and TMZ treatments revealed a significant reduction in both proteins when AE was combined with TMZ. This downregulation of MGMT and the NF-κB subunits (p50 and p65) suggests that the combined treatment may reduce drug resistance in GBM by inhibiting these key proteins [56].

In vitro, AE provided significant protection to SH-SY5Y cells against OGD/R (oxygen–glucose deprivation/reperfusion) damage and reduced inflammatory cytokine production in LPS-stimulated BV2 cells. Moreover, Western blot analyses demonstrated that AE notably increased the expression of PI3K, AKT, and mTOR proteins. Additionally, AE significantly reduced NF-κB protein levels in BV2 cells. However, when the AKT-specific inhibitor MK-2206 2HCL was used to block AKT expression, it prevented the protective effect of AE on SH-SY5Y cells subjected to OGD/R injury [57].

In recent studies, the proliferation of U87MG cells at different concentrations of aloe emodin (AE) (20 and 40 μM) over varying time intervals (24, 48, and 72 h) was examined. The results showed that both concentrations inhibited cell growth as early as 24 h into treatment, with the highest inhibition observed at 48 and 72 h, where approximately 50% of cell growth was suppressed. Moreover, it is suggested that AE not only inhibits glioblastoma cell proliferation but also induces a cell cycle arrest in U87MG cells, confirmed by FACS analysis. The data revealed that prolonged exposure to AE (20 and 40 μM) caused an accumulation of cells in the S and G2/M phases, thus arresting the cell cycle in these phases. To understand this mechanism entirely, the expression of p53, p21, and cyclin CDK2 proteins was analyzed. The results showed that AE consistently raised the levels of p53 and p21 proteins, which are involved in regulating cell cycle progression. Meanwhile, CDK2 expression, which is active in the S phase, decreased only after 48 h of treatment. Therefore, it was revealed that AE-induced cell cycle arrest is linked to increased p53 and p21 expression and decreased CDK2 expression. To sum up, AE in U87MG cells reduces cell proliferation and blocks the cell cycle at the S and G2/M phases. Additionally, the role of MAPKs in regulating cell growth was investigated, as well as the phosphorylation of Akt serine in U87MG cells treated with AE (20 and 40 μM) for short durations (2 and 4 h). A reduction in phosphorylation was observed, which may contribute to the inhibition of proliferative signals. The pro-apoptotic effects of AE from the microscopic observation of treated cells were also explored. The pro-apoptotic effect of AE was further confirmed through Western blot analysis, which showed a decrease in the normal form of PARP1 (116 kD) in treated cells and the activation of Lamin A compared to the control [58].

AE was also identified as a trigger for pyroptosis. Additionally, it was found that, when used at its IC_50_ concentration (48.7 µM for U87MG cells), AE significantly suppressed the activity of U87 cells after 48 h. Additionally, it was shown that higher concentrations lead to pyroptosis in GBM cells, while lower concentrations did not produce the same effect. Moreover, recent studies further revealed that delivering AE via nanoparticles (AE-NPs) enhances its accumulation in GBM tissue. This concentration difference allows for the effective induction of pyroptosis in tumor cells while reducing the likelihood of pyroptosis in normal brain tissue, a key factor in minimizing the side effects typically associated with chemotherapy. By incorporating a pH-sensitive and blood–brain barrier-penetrating nanocarrier, we achieved improved tumor targeting, enhanced antitumor effects, better BBB penetration, and reduced toxicity [59].

It has been proposed that AE significantly reduces oxidative stress in scopolamine-treated hippocampal tissue, providing protection to the nervous system from oxidative damage. To further explore its antioxidant capabilities, we investigated whether AE could offer cytoprotective effects against H_2_O_2_-induced injury in PC12 cells. Due to its high membrane permeability, H_2_O_2_ is commonly used as a toxic agent in vitro to mimic oxidative stress-induced injury, as elevated levels of H_2_O_2_ are known to be harmful to neurons. In our study, a 2 h treatment with 200 µM of H_2_O_2_ led to significant cell loss, as demonstrated by MTT and LDH assays. Additionally, H_2_O_2_ exposure increased extracellular nitric oxide (NO) and intracellular reactive oxygen species (ROS). However, treatment with AE markedly reduced NO and ROS levels to those similar to the control group, suggesting that AE effectively inhibits both the release of NO and the buildup of intracellular ROS caused by H_2_O_2_ in PC12 cells. In conclusion, AE was able to mitigate oxidative stress induced by H_2_O_2_, and its protective effect was largely linked to the reduction in ROS production [60].

AE demonstrated the strong inhibition of inflammatory factors associated with BV2 cells activated by LPS and provided significant protection against OGD/R-induced damage in SH-SY5Y cells during in vitro testing. Further mechanistic studies revealed that AE’s antioxidant and anti-inflammatory properties were regulated via the PI3K/AKT/mTOR and NF-κB pathways. Specifically, AE reduced the production of inflammatory cytokines in LPS-stimulated BV2 cells, as shown by decreased levels of NO and IL-6 and the downregulation of NF-κB and TNF-α expression, as indicated by Western blot analysis. The small molecule inhibitor MK2206 2HCl was found to reduce the protective effects of AE on SH-SY5Y cells exposed to OGD/R, emphasizing the role of the PI3K/AKT pathway in AE’s neuroprotective activity. Overall, these results highlight AE’s potential as a neuroprotective agent with both anti-inflammatory and antioxidant effects, making it a promising candidate for further study in conditions involving oxidative stress and neuroinflammation [57].

**Table 3:** Anticancer activity of aloe emodin and physcion in in vitro models of nervous system cancers.

Compound	Model	Cell Line	Mechanism of Action	Dose	References
Aloe emodin	In vitro monotherapy and polytherapy with TMZ (temozolomide)	Primary human GBM cells—NULU, ZAR	↓ cell growth and viability	20 µM or 20 µM + 10 µM TMZ	[56]
Aloe emodin	In vitro monotherapy	U87MG	Cell cycle arrest in S and G2/M, proapoptotic effects, ↑ levels of p53, p21, ↓ CDK2, ↓ phosphorylation of Akt serine,	20–40 µM	[58]
Aloe emodin	In vitro monotherapy in nanoparticles	U87MG, GBM mouse cell line GL261	↓ Cell proliferation ↑ pyroptosis	50–100 µM	[59]
Aloe emodin	In vitro monotherapy	BV2 cells, MK-2206 2HCL	↓ inflammatory cytokine production, ↑ expression of PI3K, AKT, and mTOR proteins, ↓ NF-κB protein levels	N/A	[57]
Aloe emodin	In vitro monotherapy	PC12 cells	↓ NO and ROS levels	40 µg/mL	[60]
Aloe emodin	In vitro monotherapy	SH-SY5Y human neuroblastoma cells, BV2 cells	Protection against OGD/R-induced damage, ↓ production of inflammatory cytokines	6, 8, 10 μM	[57]
Physcion	In vitro monotherapy	SK-N-BE92)-C cells	↑ hST8Sia VI mRNA levels, ↑ ERK, JNK, and p38 MAPK	40 µM	[61]

### 4.3. Physcion

The latest studies provide the first evidence of transcriptional regulation of the *hST8Sia VI* gene. It was demonstrated that the hST8Sia VI expression was upregulated in SK-N-BE(2)-C cells upon physcion stimulation, with the Pax-5 binding site between positions −262 and −256 playing a key role in this transcriptional activation. Physcion treatment increased *hST8Sia VI* mRNA levels in these cells. A 2.6 kb region upstream of the hST8Sia VI transcription start site was isolated, which lacks typical TATA and CCAAT boxes but contains various putative regulatory elements. It was shown that the functional promoter responding to physcion is located in the 5′-flanking region (pGL3-2660) of the *hST8Sia VI* gene, with the core promoter between positions −574 and −240, containing NF-Y and Pax-5 binding sites. Through site-directed mutagenesis and ChIP assays, it was confirmed that the Pax-5 binding site is critical for transcriptional activation by physcion. It was also found that physcion activated ERK, JNK, and p38 MAPK signaling pathways in SK-N-BE(2)-C cells, with the ERK and p38 pathways mediating the transcriptional activation of hST8Sia VI (Table 3) [61].

### 4.4. Chrysophanol

Studies indicate that chrysophanol has a protective effect against glioma by reducing the viability of U251 and SHG-44 cells and inducing dose-dependent apoptosis (Table 4). Flow cytometry analysis of the cell cycle revealed that chrysophanol treatment resulted in cell cycle arrest in the G1 phase, as indicated by a significant increase in the number of cells in this phase. Additionally, using Western blot analysis, it was observed that the protein expressions of cytosolic cyt C, cleaved caspase-3, and cleaved caspase-9 were markedly increased, indicating their role in promoting apoptosis by upregulating these proteins. Conversely, the expressions of cytosolic cyclin D1 and cyclin E in U251 and SHG-44 cells were significantly decreased, suggesting a downregulation of these cyclins. Additionally, the experiments demonstrate that chrysophanol induces the accumulation of ROS in the mitochondria of glioma cells, triggering the release of cyt C from the mitochondria into the cytoplasm and thereby promoting apoptosis in these cancer cells. Using MitoTempo (mitochondria-targeted antioxidant), the positive role of chrysophanol in glioma mitochondrial apoptosis was confirmed. It was proven that it partially reverses the effect of chrysophanol on mitochondrial ROS accumulation, which induces cell apoptosis and promotes cyt C leakage into the cytosol in glioma cells [62].

In vitro, chrysophanol dose-dependently reduced the proliferation of HBL-52 meningioma cell line. These results were based on observations of decreased cell viability, colony formation, and bromodeoxyuridine incorporation. The studies showed that chrysophanol may induce Bax expression and inhibit Bcl-2 levels, leading to a shift in the Bcl-2/Bax ratio—a key factor determining apoptosis induction—toward cell death. Treatment with chrysophanol notably increased the levels of cleaved caspase-3 and caspase-9, as well as the activities of caspase-3 and -9. Moreover, chrysophanol caused cell cycle arrest in the G1 phase and inhibited the OGN/mTOR signaling pathway but activated the neurofibromatosis 2 (NF2) pathway. The overexpression of OGN (osteoglycin) activated mTOR, downregulated NF2, and partially reversed the growth inhibition caused by chrysophanol [63].

Recent studies indicate that chrysophanol, at concentrations of 25 to 100 µmol/mL, significantly inhibits the proliferation of SH-SY5Y neuroblastoma cells, reducing cell viability from 84.8% to 72.0% after 24 h and from 96.4% to 50.0% after 48 h of incubation. Notably, chrysophanol appears to counteract the cytotoxic effects of 5-(4-N, N-diacetoxylphenyl)-10,15,20-tetraphenylporphyrin (DTPP)-mediated photodynamic therapy (PDT), likely by scavenging the ROS generated during PDT. Furthermore, chrysophanol provides some level of membrane protection post-PDT at concentrations of 6.5 to 50 µmol/mL, although this effect was dose-independent, indicating that the compound may target cell membranes as part of its protective mechanism [64].

**Table 4:** Anticancer activity of chrysophanol in in vitro models of nervous system cancers.

Coumpound	Model	Cell Line	Mechanism of Action	Dose	References
Chrysophanol	In vitro monotherapy	U251 and SHG-44	↓ cell viability, induction of apoptosis, G1 phase cell cycle arrest, ↑ apoptosis-related proteins, ↓ cell cycle proteins level	20, 50, and 100 μM	[62]
Chrysophanol	In vitro monotherapy	SH-SY5Y	↓ cell viability	25 µmol/mL to 100 µmol/mL	[64]
Chrysophanol	In vitro monotherapy	HBL-52	↓ cell growth, ↑ apoptosis, altered levels of p-mTOR and NF2, and cell growth by changing OGN expression	15–90 µM	[63]

### 4.5. Hypericin

Photodynamic therapy (PDT) with various photosensitizers is a valuable treatment approach for cancers, including those involving the nervous system [65]. The mechanism of PDT is based on the interactions between three components: chemosensitizer, proper wavelength light, and oxygen dissolved in the cell. Upon entering the cell, the photosensitizer is irradiated and transitions from its ground singlet state to an excited singlet state. After emitting some energy as fluorescence, it reaches the excited triplet state (T1). From this moment, two mechanisms can occur. The first one is based on the transfer of hydrogens or electrons between the T1 state sensitizer and the surrounding tissues (substrate), which results in the formation of free radicals and anion radicals of the sensitizer and the substrate. The further interaction of electrons with oxygen starts a cascade of production of reactive oxygen species in the cell, finally leading to its destruction. The second mechanism is based on a direct transfer of energy from T1 to the oxygen molecules. Generated singlet oxygen particles have strong oxidizing properties. Usually, both mechanisms are involved in cancer cell destruction [66]. Among natural photosensitizers, Hyp stands out as especially promising due to its strong fluorescent properties. The hydrophobic nature of Hyp enables it to permeate cell membranes, leading to its accumulation primarily within cellular organelles, such as the mitochondria, endoplasmic reticulum (ER), Golgi apparatus (GA), and lysosomes. These organelles house key members of the Bcl-2 family, which serve as primary regulators of apoptosis [67]. Fluorescence lifetime imaging and the spectroscopic analysis of Hyp-induced PDT on glioma cells revealed a progression of damage, including cell shrinkage and rounding, the formation of membrane blebs, degradation of the ER, and eventual membrane destabilization, allowing external fluid to flood the cell. Consequently, cell death triggered by Hyp-induced PDT in glioma cells was predominantly apoptotic, followed by necrosis [68].

Three-dimensional tumor spheroids serve as an outstanding model system because they mimic 3D cell–cell interactions and include an extracellular matrix similar to that of in vivo tumors. The use of Hyp as a photosensitizer has shown encouraging results, as Hyp can penetrate deep into the tissue core within 30 min of incubation, inducing O_2_ activation in a dose-dependent manner [69].

Because of the hydrophobic nature of Hyp, its intravenous administration poses a problem. One of the solutions is lipid-based formulation, with a rising interest in the use of endogenous lipoproteins. Low-density lipoprotein (LDL) molecules are transported to cells via endocytosis mediated by LDL receptors, whose number is usually increased in cancer cells. This makes LDLs a promising transport system for Hyp. On the other hand, much smaller particles of high-density lipoprotein (HDL) are also of interest. It was shown that LDL is more suitable for the gradual release of Hyp, while combination with HDL results in a more rapid Hyp release [70].

Although Hyp is widely recognized as a potent photosensitizer, in vitro studies have also revealed its significant anticancer activity against nervous system tumor cells, even without light activation (Table 5). Despite this, most scientific studies have revealed that Hyp does not significantly affect the viability of U87 MG glioblastoma cells. After 24 h of incubation, cell viability decreased by only 5–7% at Hyp concentrations ranging from 1 to 10 μM, and, after 48 h, a 22% reduction was observed at a concentration of 10 μM. However, a synergistic cytotoxic effect was observed when Hyp was combined with gossypol (Goss), whereas their individual applications had markedly weaker effects. In silico modeling combined with in vitro fluorescence spectroscopy experiments using small Bcl-2 peptide segments, designed to mimic the BH3 and BH1 domains, demonstrated that Hyp interacts with these peptides in a concentration-dependent manner [71]. In a similar study, a 4 h incubation with Hyp in U87 MG cells caused notable shifts in Bcl-2 distribution patterns. There was a prominent translocation of Bcl-2 into the nuclei, with a corresponding decrease in Bcl-2 signal in areas outside the nucleus within U87 MG cells. The effect of Hyp on Bcl-2 distribution was dependent on both time and concentration. Moreover, molecular docking studies indicated that Hyp may also interact with other anti-apoptotic members of the Bcl-2 family, including BclXL and Mcl1, in a manner similar to pan-Bcl-2 BH3 mimetics like Goss and TW37 [67].

In the U87 cell line, the presence of Hyp in the dark caused slight mitochondrial hyperpolarization. Furthermore, a significant increase in ROS production was observed after 24 h of incubation with Hyp. However, this ROS level was lower than that generated by Hyp-mediated PDT in cell lines. Hyp significantly affected U87 MG cells by markedly reducing basal, proton-linked, and maximal oxygen consumption rates (OCRs), as well as the OCR/ECAR (extracellular acidification rate) ratio. This suggests a substantial shift towards increased glycolytic activity in cellular metabolism. Consequently, in U87 MG cells, Hyp appeared to decelerate overall metabolism and redirect energy production towards glycolysis. Moreover, Hyp induced notable alterations in the endomembrane system of the cell line. Compared to the control, cells treated with Hyp exhibited a more fragmented ER network with a reduced number of ribosomes. Some ER branches appeared smooth, with ribosomes sparsely distributed on their surface. Additionally, Hyp caused ultrastructural changes in the GA, characterized by swollen GA cisternae and a decreased number of vesicles in these regions [72]. The presence of Hyp in U87 MG cells also led to an increased number of lysosomes uniformly distributed throughout the cytoplasm, while the mitochondria formed a complex tubular network structure [73].

In another study, the localization of the highly hydrophobic Hyp was tracked using the time-resolved imaging of NBDC6 (a fluorescent ceramide) in U87 MG glioma cells. A decrease in the fluorescence lifetime of NBDC6 within the cells indicates that Hyp may also follow this pathway. This may affect various processes, including the inhibition of proliferation, the activation of sphingosine-1-phosphate, lipid metabolism, oxidative stress, and the drug efflux mechanism, all of which could be enhanced by the light activation of Hyp. Western blot analysis revealed an increase in PKCδ autophosphorylation at Ser645 (p(S645)PKCδ) in glioma cells treated with 500 nM of Hyp. Confocal fluorescence microscopy identified the distinct localization of p(S645)PKCδ between Golgi apparatus-associated compartments and the nucleus [74].

Recently, in contrast to the previously cited studies, in a study on the effect of Hyp on the viability of the U87 cell line, it was observed that the IC_50_ value after 24 h of incubation was 1.5 μg/mL, and the results of flow cytometry at the mentioned concentration of this compound showed that the main mechanism of cell death was apoptosis. In this experiment, gene ontology analysis showed that terms like the positive regulation of cell proliferation, response to endoplasmic reticulum stress, cytokine-cytokine receptor interaction, and growth factor activity were enriched among down-regulated genes, while extracellular matrix organization was enriched among up-regulated genes. Fisher’s exact test was applied to analyze up- and downregulated genes against microRNA and transcription factor target databases, identifying modulators with an over-representation of targets in the DEGs. For microRNAs, hsa-miR-124-3p targeted 17 downregulated genes, while hsa-miR-26b-5p targeted 15 upregulated genes. Targets of transcription factors *CTCF*, *ATF3*, *WT1*, and *MYC* were also found to be significantly enriched among the differentially regulated genes. Differentially regulated transcription factors (DETFs) and transcription factors associated with differentially regulated genes (EG-TFs) were analyzed in comparison to transcription factors known to promote stemness in glioma cancer cells. Notably, both DETFs and EG-TFs showed a significant overlap with transcription factors in GBM cancer stem cells [75].

**Table 5:** Anticancer activity of hypericin in in vitro models of nervous system cancers.

Coumpand	Model	Cell Line	Mechanism of Action	Dose	References
Hypericin	In vitro monotherapy	U87MG	↑ROS level, ↓ Bcl-2 proteins	0.5 µM	[72]
Hypericin	In vitro monotherapy	U87MG	Interaction with Bcl-2 protein	1, 5, 10, 15, and 30 µM	[71]
Hypericin	In vitro, PDT	U-87 monoclonal and polyclonal luciferase transfected cell lines (U87-luc)	↑ bioluminecscence after addition of D-luciferin	N/A	[76]
Hypericin	In vitro, PDT	U87MG	↑ Apoptosis followed by necrosis	1 μM	[68]
Hypericin	In vitro PDT, photobiomodulation (PBM)	U87MG	↑ caspase-3 and ↑ apoptosis	200 and 500 nM	[77]
Hypericin	In vitro combined therapy with rottlerin and PDT	U87 MG	↑ apoptosis, ↑ in phosphorylated Bcl-2, ↓ intracellular oxidative stress,	0.5 μM	[78]
Hypericin	In vitro, PDT	U87 MG	↑ Apoptosis, ↓ phosphorylation of pPKCδ (Tyr155) and ↑ pPKCδ (Ser645)	0.5 μM	[73]
Hypericin	In vitro monotherapy	U87 MG	↑ PKCδ autophosphorylation at Ser645 (p(S645)PKCδ)	0.5 μM	[74]
Hypericin, emodin	In vitro combined with emodin	U87 MG	↑ intracellular concentration of Hyp	10 μM each	[41]
Hypericin	In vitro, PDT	D283 Med	↑ Photocytotoxicity, LD_50_ = 0.47 J/cm^2^	2.5 μM	[79]
Hypericin	In vitro, PDT	U373 MG, LN229 and T98G	↑ Photocytotoxicity, LD_50_ = 0.15–0.20 J/cm^2^	2.5 μM	[80]
hypericin	In vitro monotherapy	U87 MG	↓ cell viability, ↑ apoptosis, impact on genes and pathways involved in cell growth, differentiation and drug resistance	1.5 μg/mL	[75]

## 5. In Vivo Studies on the Anticancer Activity of Anthracene Derivatives

A multitude of in vitro studies that were performed on the anthracene derivatives of natural origin list different molecular targets of these tricyclic molecules. However, despite a wide range of information collected from the tests in cells, the number of pre-clinical studies that are performed on animal models is scarce, and no significant increase in their number has been observed within recent years.

Most tests are performed on different forms of glioma types of cancer; however, these studies can give an insight into the potential mechanisms of action that can be observed for anthraquinones. As an example, previous studies in mice show apoptotic effects of emodin in mice with colon cancer that was achieved by G0/G1 phase cell cycle arrest and resulted in a decrease in both weight and volume of tumor [81]. In another study, the treatment of colorectal cancer with chrysophanol [82] increased the number of positive apoptotic cells in the TUNEL test, upregulated the levels of PUMA, Bax, and caspase-3, and decreased the levels of Bcl-2 protein and Ki-67 positive cells with no significant toxicological changes observed in soft organs or in the blood parameters. The anticancer activity of physcion [83] could be related to enhanced expression levels of Bax and cleaved caspase-3 but also to the suppressed expression levels of Bcl-xL, Bcl-2, Nrf2, HO-1, SOD-1 and SOD-2 proteins, suggesting an antioxidant mode of action next to a further reported immunomodulatory activity, due to the ability to regulate the levels of interleukins, interferons, and tumor necrosis factors in a way that reduces the progression of breast cancer.

Rhein—another anthraquinone derivative—was capable of inducing apoptosis and inhibiting the EGFR and STAT3 pathways in tumor remnants, in combination with EGFR inhibitors, with clear suppression of the Bcl-2 level and an increase in the level of Bax [84], and, additionally, inhibiting the expression of β-catenin [85], which was proven in a mice HepG2 xenograft model, together with the inhibition of tumor growth.

In summary, recent publications on the anticancer properties of anthraquinone derivatives list a few interesting studies that were performed on xenograft mice models. All of them, even if performed on different cancer types, underline the ability of these plant secondary metabolites to reduce the tumor volume and weight, to induce the process of apoptosis, induce the cell cycle arrest, increase the levels of caspase-3, E-adherin, Bax protein, CD8+ T cells, and serum levels of interleukins and tumor necrosis factors. Additionally, the antiradical mechanisms inside the tumor are disturbed upon the administration of anthraquinones, and the level of Bcl-2 protein is decreased. All authors state that the compounds in the tested doses did not show any toxicity to the normal cells or soft organs, which makes the anthraquinone derivatives important drug candidates that can be used in cancer treatment alone, but also, most importantly, together with other chemotherapeutics.

As mentioned above, glioblastoma treatment with anthraquinone derivatives certainly needs further studies. The scientific literature lists only a few positions; however, these can prove the potential of anthracene derivatives to be successfully implemented into the treatment of glioblastoma.

Nevertheless, in a study conducted by Pecere et al. [86], aloe-emodin was proven to exhibit special anticancer properties against the development of neuroectodermal tumors. This study was performed in female SCID mice injected with human neuroblastoma cells (IMR5), where aloe-emodin was administered immediately after the s.c. injection of tumor cells at a dose of 50 mg/kg/day for the following 15 days. This component of aloe species was found to induce apoptosis while very selective against tumor cells. No signs of intestinal, neurological, hematological, acute toxicity or structural abnormalities were noted during the treatment. According to the authors, its action was based on a specific energy-dependent way of the aloe-emodin’s incorporation into the tumor cells that explained the low cytotoxicity to the animal organism. Interestingly, the compound’s action was observed at 37 °C, whereas its administration at 4 °C did not provide any result due to an abolished aloe-emodin uptake into the cells. To confirm this energy-based theory, the authors performed further trials on ATP-depleted cells where no cyctotoxic effect was observed. Based on these results, it may be concluded that the transport of a drug into the neuroectodermal tumor cells is unlikely to be based on passive diffusion or other membrane partition phenomena but requires physiological temperature, energized cells, and possibly a receptor-mediated recognition process, as the glucosylated form of emodin was found inactive. As a result of aloe-emodin-based therapy, apoptotic effects were observed in tumor cells, like the G0/G1 phase arrest by DNA damage (Table 6). The receptor-mediated mechanism of aloe-emodin penetration was later confirmed by the same group of researchers in vitro [87], indicating that this anthracene derivative is entering tumor cells through somatostatin receptors (SSTR2 and SSTR5).

**Table 6:** Anticancer activity of anthracene derivatives in in vivo model.

Coumpound	Model	Organism	Mechanism of Action	Dose	References
Aloe-emodin	Neuroblastoma model: neuroectoderma	SCID mice xenograft model with neuroblastoma cells (IMR5)	G0/G1 cell cycle arrest; high selectivity towards tumor cells	50 mg/kg/day from 0 to 15 days	[86]
Rhein lysinate (RL)	Glioma	Fourteen 4–6 weeks old BALB/c nude mice xenograft model with human glioma U87 cells	*↓* tumor growth by 31.9%	50 mg/kg RL i.p. from the 9th day for the following 14 days	[88]
Hypericin	Glioma	Wistar rat xenograft with glioma C6 cells	Accumulation of hypericin in the tumor cells	A single dose of 5 mg/kg b.w.	[89]
Hypericin	Recurrent malignant gliomas	42 patients after standard chemotherapy or without chemotherapy	Stable disease in 7 patients, partial response in 2 patients	An increasing dose from 0.05 to 0.5 mg/kg for 3 months, with 0.33 ± 0.07 mg/kg median dose	[90]

In another study, the anticancer properties of rhein in the form of a rhein-lysine salt (rhein lysinate—RL) expressed a significant reduction of tumor growth of 31.9% when tested in a BALB/c nude mice xenograft model with human glioma (U87) cells [88]. Among the underlying mechanisms of RL’s action tested parallel in vitro was the proliferation blockage at the G1/S phase of the cell cycle, the increase in the reactive oxygen species levels in the tumor, the upregulation of Bax and Bim proteins, and the upregulation of Bcl-2 and cyclin D1 protein.

Certainly, the most interesting compound from the anthracene derivatives, a promising candidate for an anticancer drug that can be used in neurological cancers is hypericin. This naphtodianthrone type of molecule differs structurally from its colleagues from the same group of metabolites. Due to a larger molecular weight and a dimeric structure of Hyp present in St. John’s Wort is characterized by different pharmacological properties [91]. The well-established applications of Hyp include its antidepressant action via monoamine oxydase A inhibition and antimicrobial or wound-healing properties. Its administration has been limited due to the evoked adverse effects. Hyp and alcoholic extracts from St. John’s Wort were proven to show photosensitizing properties, which, at the beginning, were perceived as serious side effects to the antidepressant treatment; however, with time and further studies, they have been found to provide an excellent capacity for neuroprotective, antimicrobial, antiviral and anticancer action. As a result of a targeted photodynamic therapy with hypericin, an induced cell death, or an inhibition of the cell cycle progression, may be achieved. The molecule, due to its high lipophilicity, can penetrate the brain–blood barrier well, which, together with low toxicity to healthy cells, makes it an interesting target of further studies [92,93].

The pioneering results on the application of Hyp in glioma were published in 2010 by Noell and co-investigators [89]. The first observations on the naphtodianthrone’s capacity in Wistar rats with implanted C6 glioma cells showed the ability of Hypat a dose of 5 mg/kg b.w. (i.v.) to penetrate the zone in healthy brain that surrounds the tumor. In postmortem studies, the accumulation of the compound was found to be higher in glioma cells with the fluorescence intensity ratio of 6.1:1 between tumor and normal tissue. Moreover, the infiltration zones in the tumor and normal tissue were 1.4:1. In the end, it was found that this compound can be an excellent glioma marker for the detection of this type of cancer in vivo after a systemic administration, with the maximum accumulation after 24 h. These initial studies drew attention to the possible applications of Hyp in glioblastoma treatment. In the following year, Coldwell et al. [90] tested synthetic Hyp in a ½ phase open-label, sequential dose escalation/de-escalation tolerance study in 42 patients with recurrent malignant gliomas (glioblastoma and anaplastic astrocytoma). Increasing doses of Hyp (0.05 to 0.5 mg/kg) were administered orally for up to 3 months, and the tumor growth was controlled on the 3rd month by magnetic resonance imaging. As a result, a slight reduction or stabilization of tumor was noted in seven patients, whereas two patients’ partial response to the treatment was observed.

These results should be revisited because, currently, and, in the next decade, a substantial increase in the development of formulation techniques is observed. Newly elaborated data on nanoforms and new delivery methods of different drugs, also of natural products, have been developed, handling new tools to deliver Hyp together with other active components directly to the tumor side.

## 6. 1,8-Dihydroxyanthraquinone Derivatives as Chemosensitizers Towards Known Chemotherapeutics

Derivatives of anthraquinones have been shown to interact with chemotherapeutic agents, often enhancing their toxic effects on cancer cells, while the chemosensitizing properties of 1,8-dihydroxyanthraquinones have been demonstrated in other types of tumors [94,95,96]. The literature on the combined use of anthraquinone derivatives and standard chemotherapeutics in treating NSCs remains limited. The results of such studies are summarized in Table 7.

TMZ is a commonly used drug in glioblastoma (GBM) treatment. However, approximately half of the patients develop resistance to TMZ. The best recognized mechanism behind it is the activity of O6-methylguanine DNA methyltransferase (MGMT) [97]. The combined use of TMZ and aloe-emodin (AE) has been shown to overcome TMZ resistance in patient-derived NULU and ZAR glioblastoma cell lines. This effect was associated with decreased MGMT protein expression, leading to a reduction in cell growth by up to 65% compared to TMZ alone. The downregulation of MGMT was likely linked to the reduced expression of the transcription factor NF-κB, as indicated by the lowered levels of its subunits, p50 and p65. Glioblastomas are known for their strong tendency to recur in a more drug-resistant state, even at sites distant from the original tumor. Scratch test results demonstrated that AE + TMZ treatment significantly reduced the migratory capacity of NULU and ZAR cells, a result not achieved with TMZ alone. Furthermore, colony formation assays revealed that, while TMZ alone had minimal impact on the clonogenic potential of GBM cells, the combination of TMZ and AE markedly reduced the number of cell colonies [56]. In another study, the combination of TMZ and Hyp in A172 and LA567 human GBM cell lines demonstrated a more cytotoxic effect than either agent used alone. The TUNEL assay and DNA laddering analysis confirmed an increased rate of apoptosis with the combined treatment. Moreover, the drug combination led to a reduction in the expression of the anti-apoptotic protein Bcl-2 and decreased cdk1–cyclin B complex activity. The synergistic effect of TMZ and Hyp was also observed in vivo in a mouse model, as indicated by slower tumor growth, higher apoptosis rates, and reduced Bcl-2 production [98].

Cisplatin (cis-diamine-dichloroplatinum) is the first metal-based anticancer drug. Its mechanism of action involves mainly producing DNA adducts, arresting DNA replication, mRNA, and protein production. Although used for a wide range of cancer (lung, gastric, ovarian, bladder), the application of the drug is limited due to some severe side effects and drug resistance [99]. Therefore, the use of aloe-emodin as an cisplatin adjuvant was tested. Surprisingly, the application of AE to cisplatin-treated C6 glioma cells exhibited a protective effect, reducing the cytotoxicity of the chemotherapeutic agent. Moreover, a combined use of cisplatin and AE considerably decreased cisplatin-induced the DNA fragmentation (apoptosis) rate and LDH release (sign of necrosis), compared to the sole use of cisplatin. AE was also evidenced to block cisplatin-triggered extracellular signal-regulated kinase (ERK) activation, and, hence, caspase activation and the initiation of apoptosis [100].

**Table 7:** Effect of combined use of 1,8-dihydroanthraquinone derivatives and chemotherapeutics in nervous system cancer settings.

Type of Cancer	Compound	Chemotherapeutic	Model	Effect	Reference
Glioblastoma	aloe-emodin (20 μM)	temozolomide (10 μM)	in vitro, primary human GBM cells NULU and ZAR	↓48–65% cell growth; ↓resistance to temozolomide (=↓expression of the MGMT protein); ↓expression of *NF-κB* transcription factor units; ↓cell migration and colony formation	[56]
Glioblastoma	hypericin (10 μM)	temozolomide (1 mM)	in vitro, A172 and LA567 cell lines in vivo, male athymic nu/nu mice injected with U87 GBM cells	↑ cell growth inhibition; ↑ cytotoxicity; ↓CDK activity; ↑ apoptosis; ↓expression of Bcl-2 protein; lower increase in tumor volume; ↑ apoptosis; ↓expression of Bcl-2 protein	[98]
Glioma	aloe-emodin (20 μM)	cisplatin (20 μM)	in vitro, C6 glioma cell line	↓cytotoxicity; ↓DNA fragmentation; ↓ LDH release; blockade of ERK activation	[100]

## 7. Conclusions

The use of natural compounds in the treatment of NSC offers significant potential due to their multifaceted mechanisms of action as well as lower toxicity profiles than traditional chemotherapeutics. While challenges such as bioavailability and resistance remain, advances in drug delivery and combination therapies may enhance the clinical utility of these compounds.

1,8-Dihydroxyanthraquinones are a class of organic compounds characterized by the anthraquinone structure, consisting of a three-ring aromatic system with a carbonyl group (C=O) at positions 9 and 10, and hydroxyl groups (-OH) at positions 1 and 8 on the anthraquinone backbone. This specific structural arrangement is of significant interest in medicinal chemistry due to its bioactive properties, including its anticancer, antioxidant, and anti-inflammatory effects. Therefore, our review aimed to provide a comprehensive overview of 1,8-dihydroxyanthraquinones and their derivatives (emodin, aloe-emodin, chrysophanol, hypericin, rhein, and physcion) highlighting their potential as promising drug candidates for NSC treatment. This manuscript also explored potential synergistic interactions between these natural compounds and existing chemotherapeutic agents (cisplatin, temozolomide) in both in vitro and in vivo settings.

It was demonstrated that 1,8-dihydroxyanthraquinone derivatives effectively inhibited NSC cell proliferation and migration, induced apoptosis, induced cell cycle arrest, reduced NO and ROS levels, and decreased Notch intracellular domain activity, non-phosphorylated β-catenin and phosphorylated STAT3 protein expression, the activation of PI3K/mTOR/GSK3β signaling pathway, and the EMT process.

The literature evidence strongly supports the potential of 1,8-dihydroxyanthraquinones as natural compounds with therapeutic efficacy in NSCs. These findings underscore their significance as promising candidates for further investigation and development in the context of nervous system cancer treatment.

## Figures and Tables

**Table 1:** Key diagnostic genes/molecular profiles altered in selected primary central nervous system (CNS) tumors (*ATRX*—alpha-thalassemia intellectual disability X-linked, *CDKN2A/B*—cyclin-dependent kinase inhibitor 2A/B, *CIC*—capicua transcriptional repressor, *EGFR*—epidermal growth factor receptor, *IDH*—isocitrate dehydrogenase, *FUBP1*—far upstream element binding protein 1, *TERT*—telomerase reverse transcriptase, *TP53*—tumor protein p53, *TSC*—tuberous sclerosis complex, *NOTCH1*—neurogenic locus notch homolog protein 1, and *NF-1*—neurofibromatosis type 1) [12].

Cancer Type	Altered Genes/Molecular Profiles
Astrocytoma, IDH-mutant	*IDH1*, *IDH2*, *ATRX*, *TP53*, *CDKN2A/B*
Oligodendroglioma, IDH-mutant, and 1p/19q-codeleted	*IDH1*, *IDH2*, 1p/19q, *TERT promoter*, *CIC*, *FUBP1*, *NOTCH1*
Glioblastoma, IDH-wildtype	*IDH*-wildtype, *TERT* promoter, chromosomes 7/10, *EGFR*
Pilocytic astrocytoma	*KIAA1549-BRAF*, *BRAF*, *NF1*
High-grade astrocytoma with piloid features	BRAF, NF1, ATRX, CDKN2A/B (methylome)
Pleomorphic xanthoastrocytoma	*BRAF*, *CDKN2A/B*
Subependymal giant cell astrocytoma	*TSC1*, *TSC2*

## Data Availability

No new data was created and analyzed in this manuscript. Data sharing is not applicable.
